# Supplementary material for: Results of the First Folate Receptor Alpha Testing Trial by the German Quality Assurance Initiative in Pathology (QuIP®)
Source: Cancers (Basel). 2025 Nov 19;17(22):3703. doi: 10.3390/cancers17223703 (PMC12651003; doi:10.3390/cancers17223703)
Supplement: Supplementary file 1 [file cancers-17-03703-s001.zip › cancers-3961282-supplementary.pdf]

Figure S1

Sensitivity of different antibodies

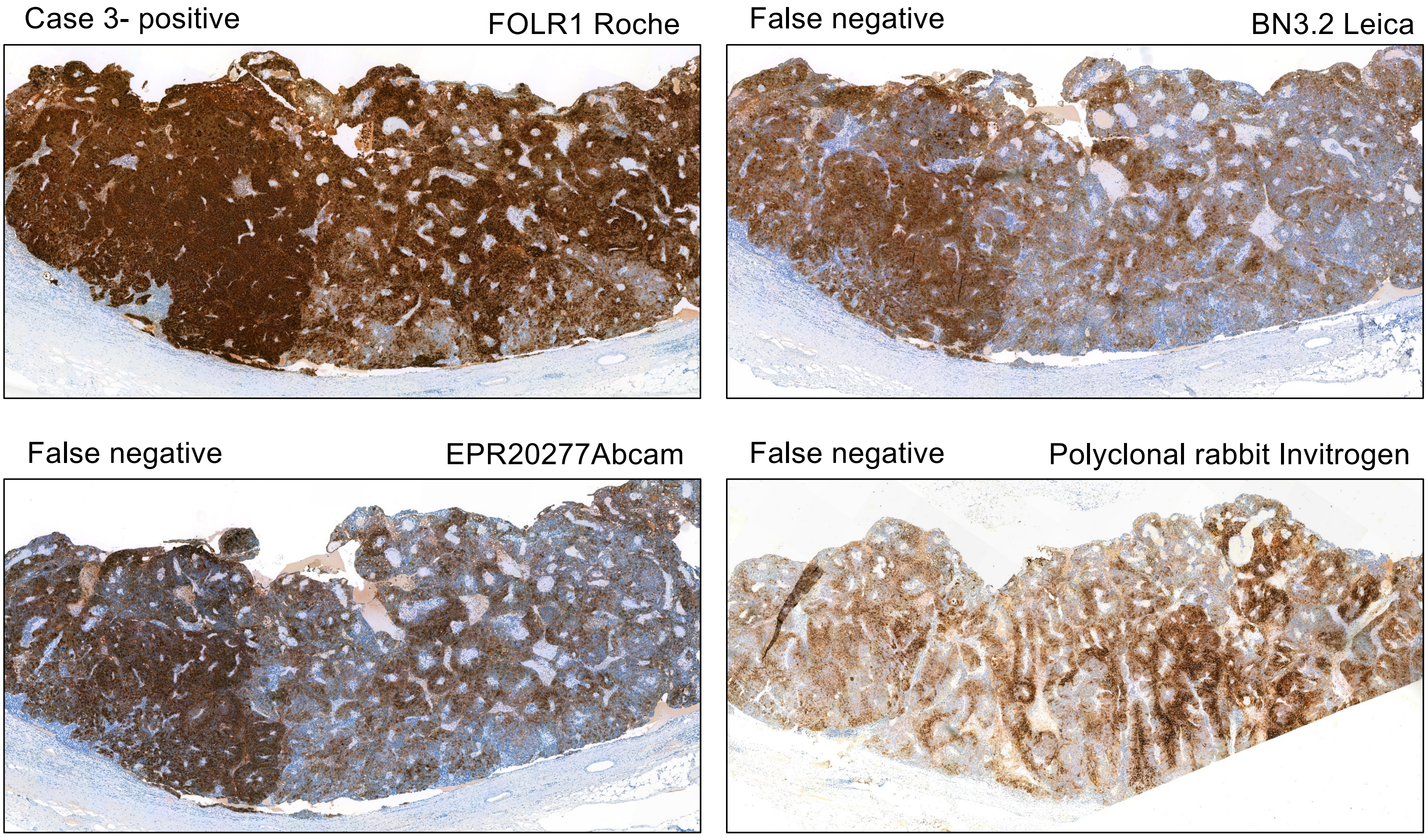

Figure S2

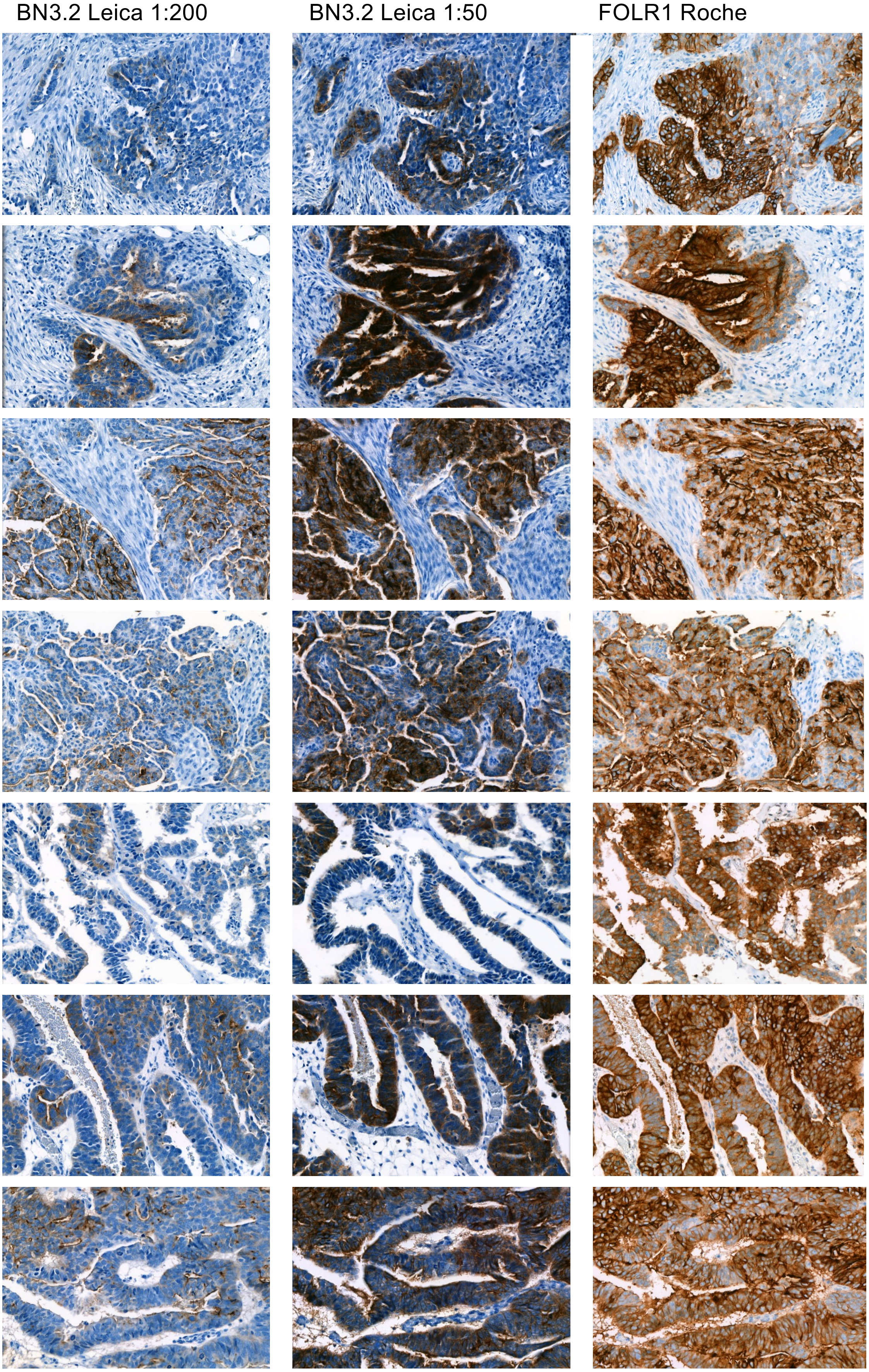

Figure S3

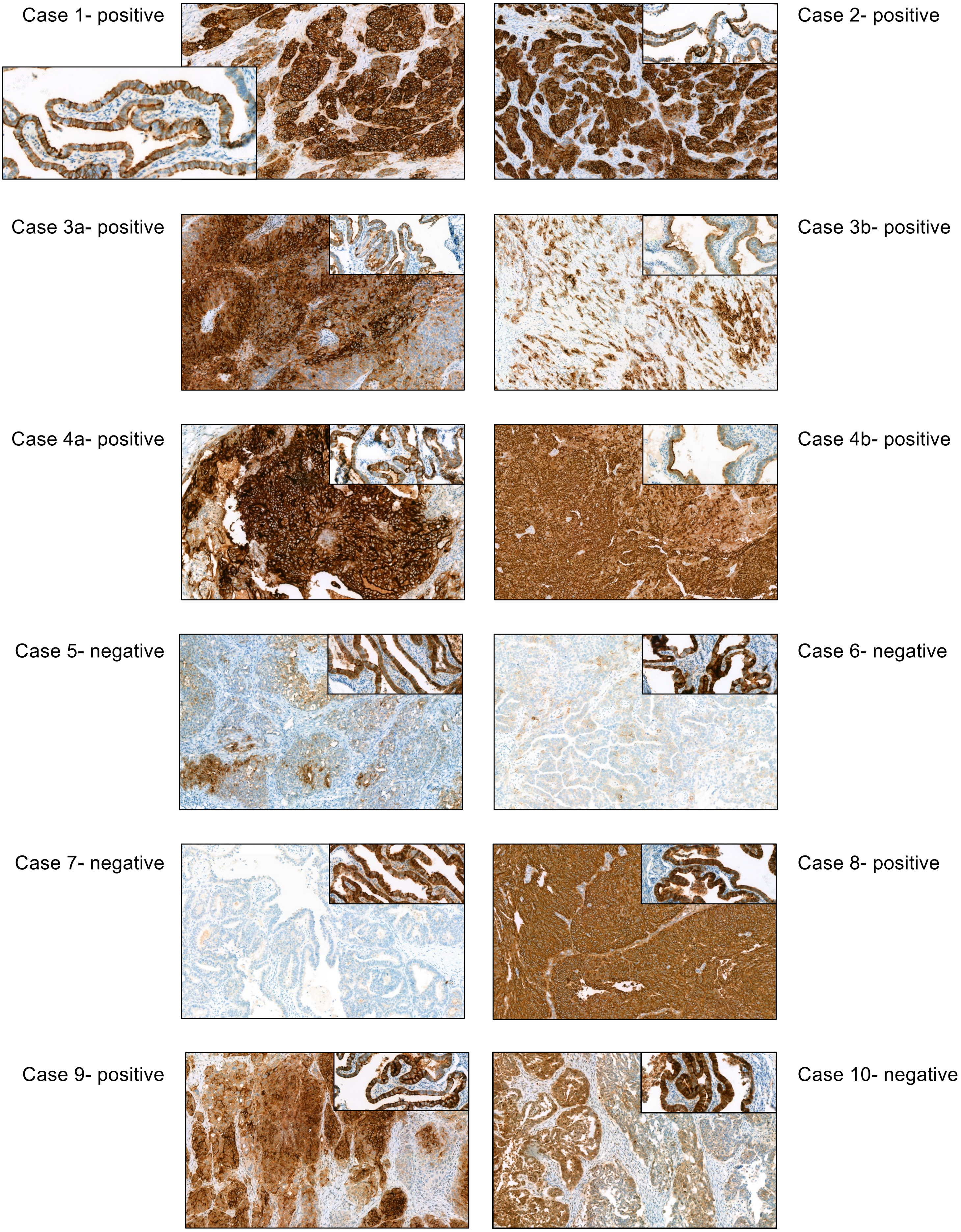

All panels: FOLR1 Roche; insets: on-slide tubal epithelium positive controls

Supplementary Table S1: Overview of internal ring trial cases not selected for open ring trial.

Internal Ring Trial identifier			Case 01		Case 02		Case 07		Case 08	
**Institute**	**Protocol**		Category	% tumor cells positive	Category	% tumor cells positive	Category	% tumor cells positive	Category	% tumor cells positive
LP1	FOLR1 (FOLR1-2.1) RxDx Assay (Ventana/Roche)	IVD	negative	10	negative	25	negative	5	negative	10
LP2	FOLR1 (FOLR1-2.1) RxDx Assay (Ventana/Roche)	IVD	negative	n.d.	negative	n.d.	negative	n.d.	negative	n.d.
P1	BN3.2 (Novocastra)	LDT	negative	60	positive	80	negative	20	negative	5
P2	26B3.F2 (BioCare)	LDT	positive	n.d.	positive	n.d.	positive	n.d.	negative	n.d.
P3	Polyclonal rabbit (Invitrogen)	LDT	positive	80	positive	80	negative	0	negative	0
P4	BN3.2 (Leica)	LDT/IVD	negative	40	negative	40	negative	15	negative	0
P5	FOLR1 (FOLR1-2.1) RxDx Assay (Ventana/Roche)	IVD	negative	10	negative	60	negative	10	negative	15

Internal Ring Trial identifier			Case 09		Case 12		Case 14		Case 15	
**Institute**	**Protocol**		Category	% tumor cells positive	Category	% tumor cells positive	Category	% tumor cells positive	Kategorie	% tumor cells positive
LP1	FOLR1 (FOLR1-2.1) RxDx Assay (Ventana/Roche)	IVD	negative	30	negative	0	negative	10	positive	80
LP2	FOLR1 (FOLR1-2.1) RxDx Assay (Ventana/Roche)	IVD	negative	n.d.	negative	n.d.	negative	n.d.	positive	80
P1	BN3.2 (Novocastra)	LDT	negative	30	negative	0	negative	0	positive	80
P2	26B3.F2 (BioCare)	LDT	positive	n.d.	negative	n.d.	negative	n.d.	positive	n.d.
P3	Polyclonal rabbit (Invitrogen)	LDT	negative	30	negative	0	negative	0	negative	55
P4	BN3.2 (Leica)	LDT/IVD	negative	40	negative	0	negative	0	negative	50
P5	FOLR1 (FOLR1-2.1) RxDx Assay (Ventana/Roche)	IVD	negative	40	negative	0	negative	20	positive	90

0

Internal Ring Trial identifier			Case 16		Case 18		Case 20		Case 21	
**Institute**	**Protocol**		Kategorie	% tumor cells positive	Category	% tumor cells positive	Category	% tumor cells positive	Category	% tumor cells positive
LP1	FOLR1 (FOLR1-2.1) RxDx Assay (Ventana/Roche)	IVD	negative	70	negative	15	negative	15	negative	20
LP2	FOLR1 (FOLR1-2.1) RxDx Assay (Ventana/Roche)	IVD	negative	n.d.	negative	n.d.	negative	n.d.	negative	n.d.
P1	BN3.2 (Novocastra)	LDT	positive	80	negative	5	negative	5	negative	0
P2	26B3.F2 (BioCare)	LDT	negative	n.d.	negative	n.d.	negative	n.d.	negative	n.d.
P3	Polyclonal rabbit (Invitrogen)	LDT	positive	80	negative	0	negative	5	negative	0
P4	BN3.2 (Leica)	LDT/IVD	negative	40	negative	1	negative	1	negative	0
P5	FOLR1 (FOLR1-2.1) RxDx Assay (Ventana/Roche)	IVD	positive	80	negative	30	negative	5	negative	10

Internal Ring Trial identifier			Case 22	
**Institute**	**Protocol**		Kategorie	% tumor cells positive
LP1	FOLR1 (FOLR1-2.1) RxDx Assay (Ventana/Roche)	IVD	negative	5
LP2	FOLR1 (FOLR1-2.1) RxDx Assay (Ventana/Roche)	IVD	negative	n.d.
P1	BN3.2 (Novocastra)	LDT	negative	5
P2	26B3.F2 (BioCare)	LDT	positive	n.d.
P3	Polyclonal rabbit (Invitrogen)	LDT	negative	0
P4	BN3.2 (Leica)	LDT/IVD	negative	20
P5	FOLR1 (FOLR1-2.1) RxDx Assay (Ventana/Roche)	IVD	negative	10

Supplementary Table S2: participant results

	n participants	proportion (%)
**Registrations**	70	
**Submissions**	70	100 %
**Successful submissions**	37	53 %
**Scores achieved**	**n participants**	**proportion (%)**
20/20 points	14	20 %
18/20 points	23	33 %
16/20 points	17	24 %
14/20 points	13	19 %
12/20 points	2	3 %
0/20 points	1	1 %

Supplementary Table S3: FOLR1 (FOLR1-2.1) (Roche) LDT protocols

	Result	Pre-treatment	Antibody clone	Dilution	Incubation time	Platform	Detection method
# 1	Successful (no deviation)	BOND Epitope Retrieval Solution 2 (Leica Biosystems)	FOLR1 (FOLR1-2.1) RxDx Assay (Roche)	RTU	20min	Bond-III (Leica)	BOND Polymer Refine Detection (Leica)
# 2	Successful (deviation case 10)	EnVision™ FLEX Target Retrieval Solution, High pH (Agilent)	FOLR1 (FOLR1-2.1) RxDx Assay (Roche)	RTU	30min	Dako Omnis (Agilent)	EnVision FLEX HRP DAB (Agilent)
# 3	Successful (deviation case 10)	EnVision™ FLEX Target Retrieval Solution, High pH (Agilent)	FOLR1 (FOLR1-2.1) RxDx Assay (Roche)	RTU	30min	Dako Omnis (Agilent)	EnVision FLEX HRP DAB (Dako/Agilent)
# 4	Not successful (deviation cases 3, 8, 9 - interpretation error)	BOND Epitope Retrieval Solution 1 (Leica Biosystems)	FOLR1 (FOLR1-2.1) RxDx Assay (Roche)	RTU	60min	Bond-III (Leica)	BOND Polymer Refine Detection (Leica)
# 5	Not successful (deviation cases 3, 8, 9)	BOND Epitope Retrieval Solution 2 (Leica Biosystems)	FOLR1 (FOLR1-2.1) RxDx Assay (Roche)	RTU	20min	Bond-III (Leica)	BOND Polymer Refine Detection (Leica)

Supplementary Table S4: Successful BN3.2 protocols

	Result	Pre-treatment	Antibody clone	Dilution	Incubation time	Platform	Detection method
**#1**	Successful (no deviation)	EDTA Puffer	BN3.2 (Leica)	1:100	30min	IntelliPATH (Zytomed Systems)	ZytoChem Plus (HRP) Polymer Kit (Zytomed Systems)
**#2**	Successful (no deviation))	EnVision™ FLEX Target Retrieval Solution, High pH (Agilent)	BN3.2 (Novocastra)	1:100	30min	Dako Omnis (Agilent)	EnVision FLEX HRP DAB (Dako/Agilent)
**#3**	Successful (deviation case 3)	BOND Epitope Retrieval Solution 1 (Leica Biosystems)	BN3.2 (Leica)	1:100	15min	Bond-III (Leica)	BOND Polymer Refine Detection (Leica)
**#4**	Successful (deviation case 1)	EnVision™ FLEX Target Retrieval Solution, High pH (Dako/Agilent)	BN3.2 (Leica)	1:100	30min	Dako Omnis (Agilent)	EnVision FLEX HRP DAB (Dako/Agilent)
**#5**	Successful (deviation case 3)	EnVision™ FLEX Target Retrieval Solution, High pH (Dako/Agilent)	BN3.2 (Leica)	1:100	30min	Dako Omnis (Agilent)	EnVision FLEX HRP DAB (Dako/Agilent)
**#6**	Successful (deviation case 3)	BOND Epitope Retrieval Solution 2 (Leica Biosystems)	BN3.2 (Novocastra)	1:50	15min	Bond-III (Leica)	BOND Polymer Refine Detection (Leica)
**#7**	Successful (deviation case 10)	BOND Epitope Retrieval Solution 2 (Leica Biosystems)	BN3.2 (Leica)	1:50	30min	Bond-III (Leica)	BOND Polymer Refine Detection (Leica)
**#8**	Successful (deviation case 3b)	EnVision™ FLEX Target Retrieval Solution, High pH (Dako/Agilent)	BN3.2 (Novocastra)	1:150	30min	Dako Omnis (Agilent)	EnVision FLEX HRP DAB (Dako/Agilent)
